# Blood‐Based Clinical Markers for Early Dementia Detection: Insights from AD‐DETECT‐Cohort

**DOI:** 10.1002/alz70856_103368

**Published:** 2025-12-25

**Authors:** Benard O Aliwa, Jasmit Shah, Olivera Nesic‐Taylor, Samuel Nguku, Sheila Waa, Juzar Hooker, Dilraj Sokhi, Sylvia Mbugua, Litha Musili, Rachel W Maina, Harrison Kaleli, Levi A. Muyela, Anne Njoki Gitere, Anne Nyambura Njogu, Zul Merali, Karen Blackmon, Chinedu T Udeh‐Momoh

**Affiliations:** ^1^ Brain and Mind Institute, Aga Khan University, Nairobi, Kenya; ^2^ Aga Khan University, Nairobi, Nairobi, Kenya; ^3^ Aga Khan University, Nairobi, Kenya; ^4^ Aga Khan University, The Brain and Mind Institute, Nairobi, Kenya; ^5^ Aga Khan University, Kenya, Nairobi, Kenya; ^6^ Aga Khan University Hospital, Kenya, Nairobi, Kenya; ^7^ Aga Khan University Hospital, Nairobi, Kenya; ^8^ Aga Khan University, The Brain and Mind Institute, Nairobi, Nairobi, Kenya; ^9^ Dartmouth Hitchcock Medical Center | Psychiatry Department, Lebanon, NH, USA; ^10^ Geisel School of Medicine at Dartmouth, Hanover, NH, USA; ^11^ Global Brain Health Institute, University of California, San Francisco, NC, USA; ^12^ FINGERS Brain Health Institute, Solna, Stockholm, Sweden; ^13^ Division of Public Health Sciences, Wake Forest University, School of Medicine, Winston‐Salem, NC, USA

## Abstract

**Background:**

Over 55 million people worldwide live with dementia, with more than 60% residing in low‐ and middle‐income countries. Alzheimer's disease, the most common form of dementia, accounts for 60–70% of cases. Early and accurate diagnosis remains a global challenge, necessitating novel approaches to mitigate the disease burden. Biomarkers hold significant promise in improving diagnostic accuracy and predicting disease progression. Validated biomarkers for the preclinical stages of dementia are crucial for advancing diagnosis and therapeutic strategies. We aimed to identify potential clinical markers for early dementia detection and assess their predictive accuracy in identifying high‐risk individuals.

**Method:**

We analyzed blood samples from dementia cases (*n* = 30) and controls (*n* = 75) for clinical parameters, including renal and liver function tests, lipid profiles, thyroid function tests, glomerular filtration rate (GFR), vitamin B12, and fasting glucose.

**Result:**

Dementia cases showed significantly elevated high‐density lipoprotein (HDL), free thyroxine (FT4), and vitamin B‐12 (*P* = 0.0002, *P* = 0.015, and *P* = 0.004, respectively) compared to controls. We also observed significant reductions in GFR, free triiodothyronine (FT3), and the cholesterol‐to‐HDL ratio (*P* = 0.003, *P* = 0.0002, and *P* = 0.05, respectively). Logistic regression confirmed associations between HDL (Odds: 10.8, 95% CI: 0.34–5.01, *P* = 0.04) and FT4 (Odds: 33.78, 95% CI: 0.64–7.20, *P* = 0.028) with dementia after adjusting for age and sex. Vitamin B‐12, FT3, GFR, and the cholesterol‐to‐HDL ratio were not significantly associated with dementia (*P* > 0.05). Predictive models demonstrated strong performance (R^2^ = 0.47–0.52).

**Conclusion:**

Our findings demonstrated the potential of HDL and FT4 as blood‐based clinical markers for early detection of cognitive impairment and dementia.

